# Assessing Knowledge and Perceptions of Vitamin B12 Deficiency and Its Impact on the Nervous System

**DOI:** 10.7759/cureus.72266

**Published:** 2024-10-24

**Authors:** Zubida H Binsiddiq, Raneem B Felemban, Teyf M Althubiani, Hazim M Almalki, Yazeed A Almalki, Wesam Ahmed Nasif

**Affiliations:** 1 Department of Medicine and Surgery, College of Medicine, Umm Al-Qura University, Makkah, SAU; 2 Department of Biochemistry, College of Medicine, Umm Al-Qura University, Makkah, SAU; 3 Department of Molecular Biology, Genetic Engineering and Biotechnology Research Institute, Sadat City University, Sadat, EGY

**Keywords:** b12 deficiency, b12 testing, neurological disorders, public health education, saudi arabia

## Abstract

Introduction

Vitamin B12 is essential for neurological function and overall health and wellness. Vitamin B12 deficiency, which is globally prevalent, can lead to severe neurological disorders. The present study aimed to evaluate awareness and knowledge of Vitamin B12 deficiency and its impact on the nervous system among residents of Makkah City, Saudi Arabia.

Materials and methods

This descriptive cross-sectional study used an online questionnaire targeting adults ≥ 18 years of age residing in Makkah City. Data were collected from 400 participants and analyzed using SPSS version 27.0.1 (IBM Corp., Armonk, NY).

Results

Eighty-nine percent of participants were aware of vitamin B12 deficiency; however, only 39.6% were tested for vitamin B12 levels. Females had higher knowledge scores (median score: 9.00) than males (median: 7.00). Pregnant women had higher knowledge scores (median: 11.00) and supplement use (66.7%). Among participants ≥46 years of age, 47.4% used supplements, whereas only 16.4% of those aged 18-25 did so. No significant differences in knowledge were found across education levels. Individuals in the workforce were more likely to take supplements than students. Testing for vitamin B12 levels was linked to higher supplement use, with 43.6% of those taking supplements compared with 13.5% of those who were not tested. Participants who were aware of the neurological effects of vitamin B12 deficiency were more likely to use supplements.

Conclusion

The results of this study highlight the gap between awareness and actions regarding vitamin B12 deficiency in Makkah City. Improved public health education and increased accessibility to Vitamin B12 testing are essential to prevent deficiencies and severe neurological complications.

## Introduction

Vitamin B12, chemically known as cobalamin, is a water-soluble vitamin necessary for the synthesis of DNA, maintenance of myelin sheaths surrounding nerves for proper nerve function, and promotion of normal homocysteine metabolism in the methionine synthase pathway as a cofactor [1]. Vitamin B12 is obtained through the consumption of animal products such as meat, milk, and eggs and is stored in the liver [2]. Therefore, it can take years for an individual to develop a deficiency if their intake is poor. It is important for the general public to understand the significance of vitamin B12 in the body and to recognize the importance of which populations are at risk for developing vitamin B12 deficiency [3].

Vitamin B12 deficiency is commonly observed worldwide [4]. It has multiple causes including inadequate dietary intake and malabsorption. Although it may affect individuals of all ages, it is more prevalent among the elderly and those who consume limited amounts of animal-derived foods [5]. Vitamin B12 deficiency can lead to short- and long-term consequences including hematological, neurological, psychiatric, and other chronic illnesses [6].

Approximately 2.5% of individuals in Western countries experience a significant lack of vitamin B12, which can lead to various neurological issues. These issues, known as subacute combined degeneration, affect both the central and peripheral nervous systems and cause a range of harmful effects [7]. One of the most significant consequences is the slow breakdown of myelin, the protective coating surrounding nerve fibers, and the eventual death of neurons. Several theories have been proposed to explain its effect on the nervous system, including the potential role of propionic or methylmalonic acid, S-adenosylmethionine, and congenital vitamin B12 metabolism [8].

Vitamin B12 is indispensable to the synthesis of myelin because it provides insulation, facilitates communication between nerve cells, and produces neurotransmitters that govern the brain’s communication networks [9]. Therefore, insufficient vitamin B12 levels pose a considerable risk to overall neurological functioning. Vitamin B12 deficiency can have mild effects on the peripheral nervous system, including numbness and tingling sensations [10]. However, prolonged deficiency leads to severe central nervous system damage, affecting myelin and neurotransmitter levels, and increasing the risk of developing vascular diseases due to elevated homocysteine levels, which affect cerebrovascular health. This can result in cognitive decline and, in extreme cases, brain death [9].

Thus, studies have emphasized the importance of recognizing vitamin B12 deficiency as a significant factor in the development of neurological damage in adults and children [10]. The objective for high-risk groups prone to vitamin B12 insufficiency is to prevent deficiency in the first place [5]. However, the primary treatment for vitamin B12 deficiency involves injection of vitamin B12 supplements. It is recommended for patients with neurological or hematological symptoms. Oral intake of vitamin B12-rich foods, such as meat, eggs, cheese, and liver, may be effective unless there is an underlying malabsorptive disorder [11].

Despite these efforts, a gap remains in understanding and addressing barriers to screening for vitamin B12 deficiency in Saudi Arabia [5]. Therefore, our research aimed to evaluate the public awareness of vitamin B12 benefits, dietary sources, and knowledge about vitamin B12 deficiency and its impact on the nervous system in Makkah City, Saudi Arabia, by identifying barriers and understanding cultural, socioeconomic, and knowledge-based factors. This knowledge could benefit the research field by developing targeted public health awareness programs to identify actionable insights that could enhance early detection rates and treat deficient individuals. This study aimed to improve health outcomes and reduce the side effects of vitamin B12 deficiency in Saudi Arabia.

## Materials and methods

This cross-sectional study was conducted between April and July 2024. The study was approved by the Institutional Review Board at Umm Al-Qura University (UQU), Makkah, Saudi Arabia, under approval number HAPO-02-K-012-2024-04-2108. Data from 417 randomly selected individuals were collected using self-administered questionnaires. The sample size was calculated (https://www.calculator.net/sample-size-calculator.html) using the following equation:

$Sample\ size\ (n) = (DEFF &times; Np [1-p]/[d2/Z21] - a/2 &times; [N-1] + p &times; [1-p])$

where n is the sample size, N is the study population of Makkah City (approximately 2,185,000, General Authority for Statistics KSA, 2024), and p is the largest percentage of any community properties surveyed, which is assumed to be 50%. The hypothesized percentage of the specific outcome frequency in the population (p) may be counted as follows: 50% ± 5. The confidence limits as a percentage of 100 (absolute ±) (d) were set to 5%. Moreover, the design effect for cluster surveys was set at 1. Using this formula, the required sample size was determined, resulting in a sample of 417 participants with 95% and 5% confidence intervals, with the latter representing the minimum acceptable limit. Future research should use a larger sample size to compensate for the possible data loss.

This study included adult residents (18-46 years old) of the Mecca region who were generally in good health, regardless of their chronic disease status. Pregnant women, smokers, and individuals under 18 years of age were excluded. Data were collected using an electronic questionnaire distributed via social media platforms (WhatsApp, Twitter, and Telegram) to the general population of Mecca.

Statistical analysis

Descriptive Statistics

Demographic characteristics were summarized using descriptive statistics and the responses of the participants regarding their knowledge and behaviors related to vitamin B12. Frequency and percentage were calculated and analyzed for categorical variables including age, sex, education level, occupation, residency status, vitamin B12 testing history, supplement use, and awareness of vitamin B12 deficiency symptoms.

Scoring Knowledge of Vitamin B12

Participant knowledge of vitamin B12 was scored on a scale from 0 to 18, based on correct responses to questions regarding its importance, deficiency effects, symptoms, dietary impact(s), and medical conditions affecting its absorption. The scoring system categorized participants into low (0-6), moderate (7-12), and high (13-18) knowledge levels.

Inferential Statistics

Inferential statistics were used to explore the associations among demographic variables, vitamin B12 testing and supplement use, and knowledge scores. Fisher’s exact test was used to assess the relationships between categorical variables including sex, age group, education level, occupation, residency status, and vitamin B12 knowledge categories. The Mann-Whitney U and Kruskal-Wallis tests were used to analyze differences in knowledge scores across categorical groups, where appropriate.

Statistical significance level and software

Differences with p < 0.05 were considered to be statistically significant. SPSS version 27.0.1 (IBM Corp., Armonk, NY) was used for all statistical analyses, including descriptive statistics, knowledge level scoring, and inferential tests, to ensure robust data analysis and interpretation.

## Results

Sociodemographic characteristics of study participants

In total, 417 participants were recruited from Makkah City, Saudi Arabia. Most participants were female Saudi nationals (64% and 96.9%, respectively). Age ranged from 18 to 45 years, with the largest group being aged 18-25 (48.2%). Most participants had a bachelor’s or postgraduate degree (72.2%), followed by those with a high-school diploma (24.2%). Occupationally, students constituted the largest group (38.8%), followed by those in the workforce (31.2%). Nearly 70% of the participants were residents of Mecca. Detailed demographic information of the cohort is summarized in Table 1.

**Table 1:** Sociodemographic characteristics of study participants

		N	%
Age (years)	18-25	201	48.2%
	26-35	63	15.1%
	36-45	75	18.0%
	46 and above	78	18.7%
Sex	Female	267	64.0%
	Male	150	36.0%
Are you pregnant? (N = 261)	No	261	97.8%
	Yes	6	2.2%
Nationality	Non-Saudi	13	3.1%
	Saudi	404	96.9%
Education level	Bachelor's or postgraduate degree	301	72.2%
	High school	101	24.2%
	Intermediate stage	10	2.4%
	Primary stage	5	1.2%
Occupation	Employee	130	31.2%
	Other	119	28.5%
	Student	162	38.8%
	Worker	6	1.4%
Are you a resident of Mecca?	No	126	30.2%
	Yes	291	69.8%

Awareness and knowledge of vitamin B12 among study participants

The survey results indicated that a significant majority (89%) of respondents were aware of vitamin B12; however, only 39.6% had been tested for vitamin B12 levels. Among those tested, 54.5% had normal levels, 41.2% had below-normal levels, and 4.2% had above-normal levels. Awareness of the importance of vitamin B12 was acknowledged by 65% of participants, and 55.4% understood the effects of deficiency. Knowledge of the symptoms of deficiency varied, with 64% recognizing numbness or tingling, 69.5% being aware of concentration or forgetfulness issues, 78.7% recognizing mood changes and/or depression, and 54.4% being aware of nausea and dizziness. Factors affecting vitamin B12 levels were low, with only 8.6% recognizing the impact of a vegan diet, 9.4% understanding the effects of specific diseases, and 8.2% knowing the impact of surgery. While 41% identified food sources rich in vitamin B12, 59.8% identified seafood, 51.6% identified eggs, and 51.3% identified red meat as sources; fewer recognized legumes, chicken, vegetables, or fruits as sources. Only 30.2% knew how to prevent deficiency, and 25.4% reported taking vitamin B12 supplements (Table 2).

**Table 2:** Awareness and knowledge of vitamin B12 among study participants

		N	%
Have you ever heard of vitamin B12?	No	46	11.0%
	Yes	371	89.0%
Have you ever been tested for vitamin B12 level?	No	252	60.4%
	Yes	165	39.6%
If yes, what’s the result? (N = 165)	Above normal	7	4.2%
	Below normal	68	41.2%
	Normal	90	54.5%
Do you know the importance of vitamin B12?	No	146	35.0%
	Yes	271	65.0%
Do you know the effect of vitamin B12 deficiency?	No	186	44.6%
	Yes	231	55.4%
Do you know that the feeling of numbness or tingling of the limbs is a symptom of vitamin B12 deficiency?	No	150	36.0%
	Yes	267	64.0%
Do you know that lack of concentration or forgetfulness is a symptom of vitamin B12 deficiency?	No	127	30.5%
	Yes	290	69.5%
Do you know that mood changes and depression are symptoms of vitamin B12 deficiency?	No	89	21.3%
	Yes	328	78.7%
Do you know that nausea and dizziness are symptoms of vitamin B12 deficiency?	No	190	45.6%
	Yes	227	54.4%
Do you know that a vegan diet affects the level of vitamin B12?	No	381	91.4%
	Yes	36	8.6%
Do you know that wheat allergy (gluten), Crohn's disease, or inflammatory bowel disease affects the level of vitamin B12?	No	378	90.6%
	Yes	39	9.4%
Do you know that gastric bypass and intestinal surgery affect the level of vitamin B12?	No	383	91.8%
	Yes	34	8.2%
Do you know any food sources for vitamin B12?	No	246	59.0%
	Yes	171	41.0%
Choose food sources that you think are rich in vitamin B12.	Seafood	211	59.8%
	Red meat	181	51.3%
	Eggs	182	51.6%
	Legumes	127	36.0%
	Chicken	133	37.7%
	Vegetables and fruits	155	43.9%
Do you know how to prevent vitamin B12 deficiency?	No	291	69.8%
	Yes	126	30.2%
Do you take any supplements for vitamin B12?	No	311	74.6%
	Yes	106	25.4%

Knowledge scores of vitamin B12 according to sociodemographic characteristics

This study found significant differences in vitamin B12 levels across demographic groups. Younger individuals aged 18-25, 36-45, and ≥46 years had higher median knowledge scores of 8.00 (interquartile range (IQR): 6.00-10.00), 9.00 (IQR: 6.00-11.00), and 9.00 (IQR: 7.00-11.00), respectively (p = 0.007), with statistically significant differences. Females had considerably higher knowledge, with a median score of 9.00 (IQR: 7.00-10.00) compared with males, who scored 7.00 (IQR: 5.00-10.00) (p < 0.001). Pregnant women had a significantly higher median knowledge score of 11.00 (IQR: 7.00-13.00) compared with non-pregnant women (9.00; IQR: 7.00-10.00; p < 0.001). Nationality had no significant effect on knowledge scores, with non-Saudis scoring 7.00 (IQR: 4.00-10.00) and Saudis scoring 8.00 (IQR: 6.00-10.00) (p =0.179). Knowledge ratings did not differ significantly according to educational level, occupation, or residence in Mecca (p = 0.681, 0.081, and 0.330, respectively), as shown in Table 3.

**Table 3:** Knowledge scores of vitamin B12 according to sociodemographic characteristics K: Independent samples Kruskal-Wallis test; U: independent samples Mann-Whitney U test. *p < 0.05 indicates significance.

		Knowledge of vitamin B12 (0-18)		
		Median	IQR	p-value, K/U
Age (years)	18-25	8.00	6.00-10.00	0.007*
	26-35	7.00	5.00-9.00	
	36-45	9.00	6.00-11.00	
	46 and above	9.00	7.00-11.00	
Sex	Female	9.00	7.00-10.00	<0.001*
	Male	7.00	5.00-10.00	
Are you pregnant?	No	9.00	7.00-10.00	<0.001*
	Yes	11.00	7.00-13.00	
Nationality	Non-Saudi	7.00	4.00-10.00	0.179
	Saudi	8.00	6.00-10.00	
Education level	Bachelor's or postgraduate degree	8.00	6.00-10.00	0.681
	High school	8.00	6.00-10.00	
	Intermediate stage	8.50	5.00-11.00	
	Primary stage	9.00	8.00-9.00	
Occupation	Employee	8.00	5.00-10.00	0.081
	Other	9.00	6.00-11.00	
	Student	8.00	6.00-10.00	
	Worker	5.00	3.00-7.00	
Are you a resident of Mecca?	No	8.00	6.00-10.00	0.330
	Yes	8.00	6.00-10.00	

Vitamin B12 supplement use according to sociodemographic characteristics

The relationship between demographic characteristics and the use of vitamin B12 supplements among 385 participants from Makkah City, Saudi Arabia, was analyzed. Supplement use was significantly associated with age, with those > 46 years using the most supplements (47.4%; p < 0.001). Younger age groups indicated less utilization, including those aged 18-25 (16.4%), 26-35 (25.4%), and 36-45 years (26.7%). Supplement use was not significantly affected by sex, with females (25.5%) and males (25.3%) using supplements at similar rates (p = 1.000). Pregnancy status had a substantial impact on supplement usage: 66.7% of pregnant women reported consuming supplements compared to 24.5% of non-pregnant women (p = 0.038).

Nationality did not have a significant effect on supplement use, with 25.2% of Saudis and 30.8% of non-Saudis using supplements (p = 0.746). Supplement use did not differ substantially according to educational levels: 25.6% of those with a bachelor’s or postgraduate degree, 23.8% of high school graduates, 20.0% of intermediate-stage students, and 60.0% of primary students reported using supplements (p = 0.351). However, supplement use was strongly affected by occupation, with individuals in the workforce (27.7%) and those in other occupations (33.6%) taking supplements more frequently than students (17.3%) (p = 0.010). Residence in Mecca had no significant effect on supplement use, with 24.6% of nonresidents and 25.8% of residents consuming supplements (p = 0.903). These data indicated considerable demographic differences in vitamin B12 supplement use, notably in terms of age, pregnancy status, and occupation, suggesting potential areas for targeted education (Table 4), compared with non-pregnant women who scored 9.00 (IQR: 7.00-10.00; p < 0.001). Nationality had no significant effect on knowledge scores, with non-Saudis scoring 7.00 (IQR: 4.00-10.00) and Saudis scoring 8.00 (IQR: 6.00-10.00; p = 0.179). Knowledge ratings did not differ significantly according to educational level, occupation, or residence in Mecca (p = 0.681, 0.081, and 0.330, respectively), as shown in Table 4.

**Table 4:** Vitamin B12 supplement use according to sociodemographic characteristics F: Fisher’s exact test. *p < 0.05 indicates significance.

		Do you take any supplements for vitamin B12?				
		No		Yes		p-value, F
		N	%	N	%	
Age (years)	18-25	168	83.6%	33	16.4%	<0.001*
	26-35	47	74.6%	16	25.4%	
	36-45	55	73.3%	20	26.7%	
	46 and above	41	52.6%	37	47.4%	
Sex	Female	199	74.5%	68	25.5%	1.000
	Male	112	74.7%	38	25.3%	
Are you pregnant?	No	197	75.5%	64	24.5%	0.038*
	Yes	2	33.3%	4	66.7%	
Nationality	Non-Saudi	9	69.2%	4	30.8%	0.746
	Saudi	302	74.8%	102	25.2%	
Education level	Bachelor's or postgraduate degree	224	74.4%	77	25.6%	0.351
	High school	77	76.2%	24	23.8%	
	Intermediate stage	8	80.0%	2	20.0%	
	Primary stage	2	40.0%	3	60.0%	
Occupation	Employee	94	72.3%	36	27.7%	0.010*
	Other	79	66.4%	40	33.6%	
	Student	134	82.7%	28	17.3%	
	Worker	4	66.7%	2	33.3%	
Are you a resident of Mecca?	No	95	75.4%	31	24.6%	0.903
	Yes	216	74.2%	75	25.8%	

Association between vitamin B12 knowledge and supplement usage

This study examined various parameters associated with vitamin B12 supplementation among the participants. A substantial association was identified between having been tested for vitamin B12 levels and supplement use, with 43.6% of those tested using supplements and 13.5% of those who were not (p < 0.001). Individuals with below-normal levels took supplements at a higher rate (57.4%) than those with normal (34.4%) or above-normal (28.6%) levels (p = 0.010). Awareness of the importance of vitamin B12 and deficiency symptoms were substantially linked with supplement use, with 32.8% and 33.3% of educated individuals using supplements, respectively, compared with 11.6% and 15.6% of those who were oblivious, respectively (p < 0.001). Recognizing mood changes and depression as signs of deficiency were also significant, with 22.3% of the aware participants taking supplements compared to 37.1% of unaware participants (p = 0.006). Informed individuals were more likely to use vitamin B12 supplements (47.2%, 48.7%, and 41.2%, respectively) than uninformed individuals (23.4%, 23.0%, and 24.0%, respectively) (p = 0.004, p < 0.001, and p = 0.038, respectively). Other characteristics, such as understanding that numbness or tingling in the limbs and lack of focus or forgetfulness were symptoms, were not significantly associated with supplement use (p = 0.061 and p = 0.272, respectively). Recognizing nausea and dizziness as signs of vitamin B12 insufficiency had no significant effect on supplement use, with similar percentages for knowledgeable and naïve individuals (25.6% versus 25.3%; p = 1.000). Overall, awareness and personal health experience had a considerable impact on the likelihood of using vitamin B12 supplements (Table 5).

**Table 5:** Association between vitamin B12 knowledge and supplement usage F: Fisher’s exact test. *p < 0.05 indicates significance.

		Do you take any supplements for vitamin B12?				
		No		Yes		p-value, F
		N	%	N	%	
Have you ever been tested for vitamin B12 level?	No	218	86.5%	34	13.5%	<0.001*
	Yes	93	56.4%	72	43.6%	
If yes, what’s the result?	Above normal	5	71.4%	2	28.6%	0.010*
	Below normal	29	42.6%	39	57.4%	
	Normal	59	65.6%	31	34.4%	
Do you know the importance of vitamin B12?	No	129	88.4%	17	11.6%	<0.001*
	Yes	182	67.2%	89	32.8%	
Do you know the effect of vitamin B12 deficiency?	No	157	84.4%	29	15.6%	<0.001*
	Yes	154	66.7%	77	33.3%	
Do you know that the feeling of numbness or tingling of the limbs is a symptom of vitamin B12 deficiency?	No	120	80.0%	30	20.0%	0.061
	Yes	191	71.5%	76	28.5%	
Do you know that lack of concentration or forgetfulness is a symptom of vitamin B12 deficiency?	No	90	70.9%	37	29.1%	0.272
	Yes	221	76.2%	69	23.8%	
Do you know that mood changes and depression are symptoms of vitamin B12 deficiency?	No	56	62.9%	33	37.1%	0.006*
	Yes	255	77.7%	73	22.3%	
Do you know that nausea and dizziness are symptoms of vitamin B12 deficiency?	No	142	74.7%	48	25.3%	1.000
	Yes	169	74.4%	58	25.6%	
Do you know that a vegan diet affects the level of vitamin B12?	No	292	76.6%	89	23.4%	0.004*
	Yes	19	52.8%	17	47.2%	
Do you know that wheat allergy (gluten), Crohn's disease, or inflammatory bowel disease affects the level of vitamin B12?	No	291	77.0%	87	23.0%	<0.001*
	Yes	20	51.3%	19	48.7%	
Do you know that gastric bypass and intestinal surgery affect the level of vitamin B12?	No	291	76.0%	92	24.0%	0.038*
	Yes	20	58.8%	14	41.2%	

## Discussion

This cross-sectional study, conducted in Makkah City, Saudi Arabia, provided significant data regarding the knowledge and perceptions of the causes of vitamin B12 deficiency and its impact on the nervous system among the local population.

The awareness of vitamin B12 among the participants was relatively high, with 89% being aware of the vitamin. This level of awareness is consistent with previous studies conducted in different regions of Saudi Arabia, where awareness levels were reported to be similarly high (64%) [12]. However, despite this high awareness, only 39.6% of participants were tested for vitamin B12 levels. The gap between awareness and testing suggests a disconnect between public health education and accessibility to testing services [13].

Sex differences in knowledge and supplemental use were also observed. Females demonstrated higher knowledge scores than males, with median scores of 9.00 and 7.00, respectively. This aligns with findings from other studies in which women generally exhibited higher health literacy levels than men [14]. Interestingly, pregnant women in this study had significantly higher knowledge scores (median score: 11.00) and higher supplement usage rates (66.7%) than non-pregnant women. The increased awareness and use of supplements among pregnant women could be a result of prenatal care programs. These programs highlight the significance of vitamins and nutrients during pregnancy [15].

Age was another significant factor that influenced vitamin B12 knowledge and supplement use. Participants ≥ 46 years of age exhibited the highest usage of supplements (47.4%), which is consistent with findings reported in other studies suggesting that older individuals are more prone to use dietary supplements due to increased health concerns and preventive health behaviors, such as taking supplements or seeking diagnostic tests for deficiencies [16]. In contrast, younger individuals (18-25 years) reported lower usage (16.4%), which may reflect a lack of perceived need for or awareness of the importance of vitamin B12 [17].

The association between educational level and knowledge of vitamin B12 was less pronounced, with no significant differences across different educational levels. This contrasts with the findings from other studies that have often reported a positive correlation between higher education levels and better health knowledge [18]. The lack of significant differences in this study suggests that public health messages regarding vitamin B12 are reaching a broad audience regardless of educational level or could indicate limitations in the study’s sampling or measurement methods.

Occupation significantly influenced vitamin B12 supplement use, with those in the workforce and those in other occupations being more likely than students to take supplements. This could be due to financial differences because employed individuals may have more resources to spend on supplements than students [19]. Additionally, the high usage rates among pregnant women highlight the effectiveness of targeted health interventions in this demographic because prenatal care often includes recommendations for vitamin supplementation [20].

Vitamin B12 levels were significantly associated with supplement use, with 43.6% of those tested taking supplements compared with 13.5% of those who were not tested. Among those tested, individuals with below-normal levels were more likely to consume supplements (57.4%) than those with normal or above-normal levels. This emphasizes the importance of diagnostic testing for identifying deficiencies and guiding appropriate supplement use [21].

Awareness of the effects of vitamin B12 deficiency, such as mood changes, depression, and symptoms related to neurological health, significantly influences supplement use. Participants knowledgeable about these effects were more likely to take supplements, indicating that understanding the health implications of deficiencies can motivate preventive health behaviors [22]. This is consistent with other studies that found a link between health knowledge and proactive health behaviors [23].

Knowledge of the factors that affect vitamin B12 levels, such as diet and some medical conditions, also plays a crucial role in supplement use. Participants who were aware of the impact of a vegan diet, wheat allergy, Crohn’s disease, or gastric bypass surgery on vitamin B12 levels were significantly more likely to use supplements. This aligns with research demonstrating that the awareness of specific risk factors can lead to targeted preventive actions [24].

The present study has significant clinical implications for public health education and preventive care. The high awareness but low testing rates for vitamin B12 levels highlight the need for increased accessibility to diagnostic services and stronger encouragement from healthcare providers for routine screenings, especially for high-risk groups such as the elderly, pregnant women, and individuals with dietary restrictions or medical conditions that affect nutrient absorption. Healthcare practitioners should focus on educating patients on the critical role of vitamin B12 in neurological health and the potential long-term consequences of deficiency. Additionally, integrating vitamin B12 status checks into regular health assessments and prenatal care programs can help identify deficiencies early and ensure timely interventions through dietary modifications or supplementation.

While current guidelines from organizations like the National Institutes of Health (NIH) and the American College of Physicians (ACP) generally do not recommend routine screening for vitamin B12 deficiency in healthy adults, they do emphasize the importance of screening for individuals with risk factors, such as a history of pernicious anemia, gastrointestinal surgery, or strict vegetarian or vegan diets. Healthcare providers should be vigilant in identifying and assessing patients who may be at risk for vitamin B12 deficiency and offer appropriate screening and treatment as needed [11].

Limitations, strengths, and recommendations

The present study had several limitations, the first of which was its cross-sectional design, which limited the ability to establish causal relationships among knowledge, perceptions, and supplement use. Reliance on self-reported data may have introduced bias because participants may overestimate their knowledge or underreport their supplement use. The sample size, although adequate for initial insights, may not be representative of the broader population of Makkah City, potentially affecting the generalizability of the findings. The study did not account for potential confounding variables, such as socioeconomic status, detailed dietary habits, or the presence of other health conditions that could influence vitamin B12 levels and perceptions.

Longitudinal studies could provide valuable insights into how knowledge and perceptions evolve and influence their impact on health behaviors and outcomes. Investigating specific barriers to testing and supplement use, such as financial constraints, lack of access to healthcare facilities, and cultural beliefs, is crucial for developing targeted interventions. In addition, research should focus on the effectiveness of different educational strategies in improving knowledge and changing health behaviors related to vitamin B12 deficiency. Comparative studies across different regions and populations could further elucidate the global patterns of awareness and supplement use, contributing to more comprehensive public health strategies.

While the data did not specify the exact type of supplement used, a significant portion of participants were likely taking multivitamin supplements containing vitamin B12. However, it is possible that some participants were taking standalone vitamin B12 supplements. Future studies may benefit from collecting more detailed information on the specific types of supplements used to provide a more precise understanding of the relationship between supplement use and vitamin B12 levels.

A limitation of our study is that participants were not informed about the scoring system beforehand. This may have influenced their responses and potentially biased the results. Future studies should inform participants about the scoring system before questionnaire completion to minimize potential biases and enhance the validity of the findings.

## Conclusions

Results of the present study highlight the significant gap between awareness and action regarding vitamin B12 deficiency in Makkah City, Saudi Arabia. While the majority of residents were aware of vitamin B12, only a small percentage have been tested for deficiency or had taken supplements. This disparity underscores the need for improved public health education and increased accessibility to vitamin B12 testing to prevent vitamin B12 deficiency and its effects on the nervous system.
